# Visible and angular interrogation of Kretschmann-based SPR using hybrid Au–ZnO optical sensor for hyperuricemia detection

**DOI:** 10.1016/j.heliyon.2023.e22926

**Published:** 2023-11-28

**Authors:** Siti Nasuha Mustaffa, Nadhrah Md Yatim, Affa Rozana Abdul Rashid, Nadrah Md Yatim, Vatsala Pithaih, Nur Shahirah Sha'ari, Ahmad Razif Muhammad, Azaham Abdul Rahman, Nur Akmar Jamil, P. Susthitha Menon

**Affiliations:** a Institute of Microengineering and Nanoelectronics (IMEN), Universiti Kebangsaan Malaysia, 43600, UKM Bangi, Selangor, Malaysia; b Faculty of Science and Technology, Universiti Sains Islam Malaysia (USIM), Bandar Baharu Nilai, 71800, Nilai, Negeri Sembilan, Malaysia; c Kulim Hi-Tech Pte Ltd, No.1, Jalan Bukit Hijau 26/24, Section 26, 40400, Shah Alam, Selangor, Malaysia

**Keywords:** Kretschmann, Surface plasmon resonance, Optical sensor, FDTD simulation, Uric acid, Zinc oxide

## Abstract

Uric acid is a waste product of the human body where high levels of it or hyperuricemia can lead to gout, kidney disease and other health issues. In this paper, Finite Difference Time Doman (FDTD) simulation method was used to develop a plasmonic optical sensor to detect uric acid with molarity ranging from 0 to 3.0 mM. A hybrid layer of gold-zinc oxide (Au–ZnO) was used in this Kretschmann-based Surface Plasmon Resonance (K-SPR) technique with angular interrogation at 670 nm and 785 nm visible optical wavelengths. The purpose of this study is to observe the ability of the hybrid material as a sensing performance enhancer for differentiating between healthy and unhealthy uric acid levels based on the refractive index values from previous study. Upon exposure to 670 nm wavelength, the average sensitivity of this sensor was found to be 0.028°/mM with a linearity of 98.67 % and Q-factor value of 0.0053 ${\text{mM}}^{-1}$. While at 785 nm, the average sensitivity is equal to 0.0193°/mM with slightly lower linearity at 94.46 % and Q-factor value of 0.0076 ${\text{mM}}^{-1}$. The results have proven the ability of hybrid material Au–ZnO as a sensing performance enhancer for detecting uric acid when compared with bare Au and can be further explored in experimental work.

## 1 Introduction

Uric acid (UA) is a crucial purine metabolism product found in human blood and urine. The normal uric acid concentration in the blood serum ranges from 0.1 to 0.4 mM [1], while the normal concentration of UA in urine excretion is from 1.19 to 2.98 mmol/day [2]. When an abnormally high UA concentration, hyperuricemia occurs, it can cause serious diseases such as gout, kidney failure, and heart failure [3]. The development of a sensor for the detection of hyperuricemia can provide a non-invasive and accurate method for the early detection and monitoring of the condition [4]. Therefore, it is crucial to have a high-sensitivity sensor for detecting UA concentrations. Many types of UA sensors have been developed, such as, electrochemical [[5], [6], [7]], amperometric [8], and optical-based sensors [[9], [10], [11], [12]]. Enzymatic-based electrochemical sensors function based on the oxidation of UA in the presence of the uricase enzyme, producing allantoin and hydrogen peroxide as by-products. However, these sensors rely on the chemical oxidation process and consequently suffer from significant drawbacks, including longer detection time and low sensitivity [[13], [14], [15]]. Meanwhile, the amperometric biosensor encounters an issue in practical applications, as it requires maintaining the electrode at approximately 0.7 V. This relatively high electrode potential can result in the reaction of other biological electroactive molecules on the electrode surface [16]. This can lead to a less sensitive sensor.

To overcome these limitations, optical sensors are used due to their label-free detection, fast response and high sensitivity [17]. An example of optical sensor used is tapered optical fiber (TOF). In the research done by Singh et al., in 2020, gold nanoparticles (AuNPs) immobilized on TOF used for detection of UA recorded the highest sensitivity at 0.0131 nm/μM. In their research, the AuNPs excited the surface plasmon (SP) at the fiber surface for the localized surface plasmon resonance (LSPR) phenomenon to occur. In addition, Islam et al., 2023 developed an optical LSPR sensor consisting of dual plasmonic layers of aluminium doped zinc oxide (AZO) and gallium doped zinc oxide (GZO). The sensor had two separate resonance peaks with refractive index detection capability from 1.30 to 1.41 and high sensitivity at 10,890.35 nm/RIU [18]. Besides the mentioned optical sensor, the SPR sensor is also widely used for biosensing application [19,20].

This paper uses an optical-based sensor using Kretschmann-configured surface plasmon resonance (K-SPR) to detect different concentrations of UA. K-SPR is chosen due to its major advantage on label-free detection [21] and its real-time monitoring capability compared to other label-free methods [22,23]. Label-free detection, in this context, refers to the detection of analytes without the need for a label or a reporter molecule. The SPR technique employed in this paper is a label-free detection method that relies on the interaction between the analyte and the sensing layer on the surface of the sensor [24]. The real-time monitoring capability is particularly useful for monitoring disease progression or treatment efficacy in biomedical applications [25], making it a promising candidate for UA biosensor. SPR is an optical phenomenon where an electromagnetic oscillation happens at the metal/dielectric interface when light interacts with the surface plasmons (SPs) on the metal surface [26]. The SPR sensor consists of a glass substrate with a nanoscale-thick metal layer. The metal layer has a vital role in SPR since it has many free electrons contributing to the negative permittivity of the materials. The SPR phenomenon occurs due to these free electrons interacting with incident light [27].

As mentioned, SPR sensor in the form of Kretschmann configuration will be used in this sensing application. In this configuration, the thin metal layer is sandwiched by a sensing medium on top of it and a prism at the bottom of the thin film [28]. To excite the surface plasmon wave at the metal-dielectric interface, p-polarized light is used by illuminating it on the metal surface through the prism [29]. The excitation is important to obtain the resonance oscillation and is based on the total internal reflection at the metal-dielectric interface. A sharp dip in the resonance curve signified the SPR phenomenon has occurred and corresponds to the minimum reflectivity on the SPR curve [30]. The SPR angle at which the resonance happens relies on the material's refractive index (RI) near the metal surface [31]. Thus, a change in RI of the sensing surface will cause a shift in the incident light of the optical wavelength, indicating the analyte's presence and concentration in the sample used. Usually, the metal layer used in SPR are gold (Au) and silver (Ag) [32]. But, due to the instability of Ag, which can easily be oxidized under air exposure, leading to poor sensitivity of Ag-based SPR sensor, Au is a better option in SPR-based sensing [33].

In SPR, since the biomolecules have to be attached to the sensing layer and not directly to the metal layer, it contributes to a low-sensitivity sensor [26]. Recently, metal oxides have been widely used to increase the sensitivity of the SPR sensor. Examples of metal oxides used are zinc oxide (ZnO) [34], graphene oxide (GO) [35] and titanium dioxide ${\text{TiO}}_{2}$ [36]. In this paper, ZnO, a porous material will be used as a sensing layer on top of Au. ZnO, a II-VI semiconductor, is used because it has a high isoelectric point, making it to easily absorb biomolecules with a lower isoelectric point [26]. At the nanoscale, ZnO confers specific properties that are highly beneficial for SPR sensors. Its increased surface area provides an excellent platform for the immobilization of biomolecules due to the biocompatibility and biodegradability of ZnO. This heightened surface area increases a greater number of interactions between immobilized ligands and the biomolecules to be detected. These interactions are fundamental for enhancing the SPR signal, thus making it more sensitive [3]. Therefore, the integration of the Au–ZnO hybrid layer can significantly increase the sensitivity in uric acid detection.

A previous study by Paliwal et al. showed how ZnO is used in SPR signal enhancement for the detection of kidney waste, urea [37]. In their research, the prism/Au/ZnO layer was used and it was found that the sensor has high sensitivity at 0.039°/(mg/dl), high reusability and reproducibility for urea detection at 633 optical wavelength. However, to date, the K-SPR optical sensor using hybrid Au–ZnO as signal enhancement for uric acid sensing based on angular interrogation techniques at 670 nm and 785 nm has not been reported yet. Therefore, this paper aims to address this notable gap. In this paper, numerical analysis using Kretschmann-based SPR is used to observe the optimum parameters needed to be used for constructing a K-SPR sensor for uric acid detection with a molarity ranging from 0 mM to 3.0 mM. Since the limitation of RI for uric acid at different concentrations from already conducted research, this study aims to sense the UA for healthy level (0 mM–2.4 mM) and unhealthy levels (3.0 mM). The full-width-at-half-maximum (FWHM), angle shift, minimum reflectivity, sensitivity and quality factor (Q-factor) of the sensor will be analyzed in this work. The material layer used in this research is BK7/Cr/Au/ZnO, where the chromium (Cr) layer acts as an adhesive layer between the borosilicate glass (BK7) and Au.

## 2 Methodology

### 2.1 Numerical simulation

From the previous work done by Menon et al. it was found that the percentage difference between the experimental and simulation results of resonance angle for the detection of glucose was only in the range of 0.2–2% [38]. Hence, the numerical simulation results are reliable to be used as a reference for experimental work in the future. In this paper, the K-SPR sensor for uric acid detection is simulated by using Finite-Difference-Time-Domain (FDTD) Solution software from Lumerical Inc. This simulation is a helpful modelling technique for studying how light interacts with materials, and this simulation solves the Maxwell equations using YEE algorithms [39,40]. The conformal mesh technology (CMT) is used for mesh refinement method. This meshing algorithm is set as ‘conformal variant 1’ that is available for all materials including metal and the minimum mesh step is 0.01 nm. The maximum mesh override region is set as 0.05 nm (dx) and 0.1 nm (dy) for x and y direction respectively. The range of mesh accuracy from low to high is 1–8; and this simulation was run using high accuracy of 6. The simulation time is 1500 fs for both 670 and 785 nm optical wavelengths.

**Fig. 1:**
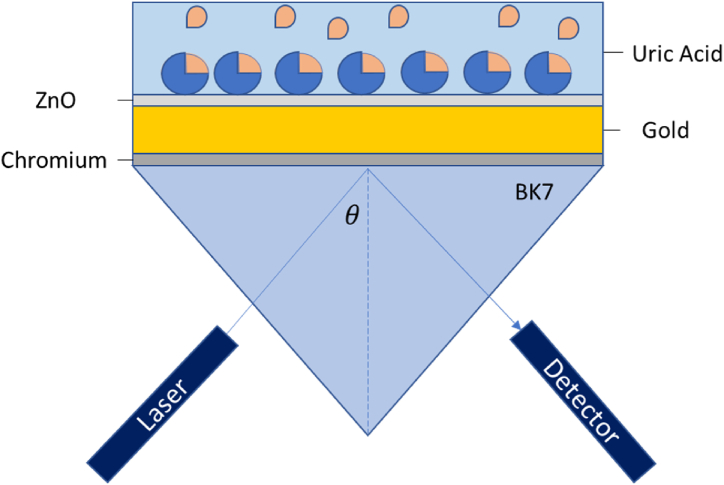
BK7/Cr/Au/ZnO K-SPR sensor for uric acid detection.

For the material layer, the geometrical position of BK7, Cr, Au, and ZnO depends on the thickness desired for the research. In this study, the x-span value for BK7 is 7950 nm, the Cr layer is 0.5 nm thick, while the thickness of Au varies from 30 to 70 nm, and the thickness of ZnO ranges from 5 to 300 nm to determine the optimum sensing layer thickness. In this study, BK7 prism was chosen to design the sensor because it is a commonly used substrate material for SPR sensor, and it provides good coupling efficiency for the incident light, due to its low refractive index [41]. The assumption made for this simulation is that the area above the sensor layers have a uniform refractive index values corresponding to air (n = 1) or the respective uric acid refractive indices. The materials configuration used in this paper is illustrated as shown in Fig. 1.

For the materials database, the refractive index of BK7, Au and Cr are taken from BioNavis [26,42] and the refractive index of ZnO is obtained from Stelling [26,43]. Meanwhile the RI of uric acid at different concentrations of 0 mM–3.0 mM were obtained from Gan et al. [11] with RI of 1.333, 1.3331, 1.3332, 1.3334, 1.3335, and 1.3336, respectively. The optical properties of the materials used are shown in Table 1. In FDTD simulation, surface plasmon excitation is observed by applying angular interrogation techniques at 670 nm and 785 nm. The choice of wavelengths 670 nm and 785 nm for the analysis in this paper is based on their common use in SPR sensing and the availability of these wavelength in BioNavis SPR Navi instrument used in the measurements [44,45]. Furthermore, research conducted by Menon et al. [46] has demonstrated that the best SPR response curve occur at 670 nm and 785 nm, as opposed to the commonly used wavelength of 633 nm. This finding has influenced this study to employ these wavelengths for uric acid sensing. The simulation utilizes the perfectly matched layer (PML) absorbing boundary conditions to absorb source light with minimum reflection [40]. Since this simulation involves metal, the conformal variant is set to 1 [47]. The background index is first set to 1.0 for air to observe the optimum layer for sensing purposes, then varied depending on the refractive index of UA at different concentrations obtained from the experiment by Gan et al. [11].

**Table 1:** Optical properties Of K-SPR sensor materials at different wavelengths.

Wavelength	633 nm		670 nm		785 nm	
Material	n	k	n	k	n	k
Borosilicate glass, BK7	1.5213	0	1.5202	0	1.5162	0
Chromium, Cr	3.3256	4.2732	3.5297	4.2685	3.9736	4.1895
Gold, Au	0.1968	3.2478	0.1741	3.6123	0.1836	4.5871
Zinc oxide, ZnO	1.6248	0.016001	1.6224	0.0050393	1.6014	0.0004098

The crucial parameters such as FWHM, resonance angle shift, minimum reflectivity ($R_{\min}$), the sensor's sensitivity and Q-factor are calculated from the K-SPR curve obtained from the simulation. The value of FWHM is important since it denotes the sensor resolution on uncertainty level in determining resonance angle and it can be calculated using Eq. (1) [12,26],

(1) $$\begin{array}{c} FWHM=\Delta \theta_{0.5} \end{array}$$

where ${\;\Delta \theta }_{0.5\;}$ is the difference in resonance angle at half width of the SPR curve. A low FWHM value of the reflectance curve is preferred because it indicates high sensor resolution with a low uncertainty in determining the resonance angle. The resonance angle shift of the SPR curve is calculated by subtracting the SPR angle of a high concentration of UA with the SPR angle of the healthy person (water). The resonance angle shift is directly proportional to the changes in the RI caused by the binding of uric acid to the sensor's surface, which has been functionalized with specific ligands. The binding process on the sensor layer will cause the RI changes, resulting in resonance angle shift in the reflected light. This parameter is crucial for determining the analyte concentration, and in this study, we focus on quantifying the concentration of uric acid to differentiate between the healthy and unhealthy UA level [48,49].

Lower minimum reflectivity denotes that the sensor can detect even small changes when the analyte binds to the sensor surface, signifying its high sensitivity. The lowest $R_{\min}$ is required since it directly influences the sensor's sensitivity [49]. Next, the sensitivity (S) of the sensor is determined by the change in SPR angle, ${\;\Delta \theta }_{SPR\;}$ per change in concentration as shown in Eq. (2) [29].

(2) $$\begin{array}{c} S=\frac{{\Delta \theta }_{SPR\;}}{change\;in\;concentration\;}=\frac{\theta_{SPR\left(uric\;acid\right)\;}-\theta_{SPR\left(water\right)\;}}{{Concentration}_{uric\;acid\;}-{Concentration}_{water\;}} \end{array}$$

The unit for the sensitivity is °/mM. Lastly, the Q-factor of the sensor is calculated using Eq. (3) [50],

(3) $$\begin{array}{c} Q-factor=\frac{S}{FWHM} \end{array}$$

where the unit for Q-factor is ${\text{mM}}^{-1}$. The Q-factor is indicative of the sharpness of the SPR curve, a critical determinant of the sensing system's sensitivity. A higher Q-factor denotes a sharper SPR curve, resulting in increased sensitivity [51]. Both high sensitivity and Q-factor indicate high sensor performance.

## 3 Results and discussion

**Fig. 2:**
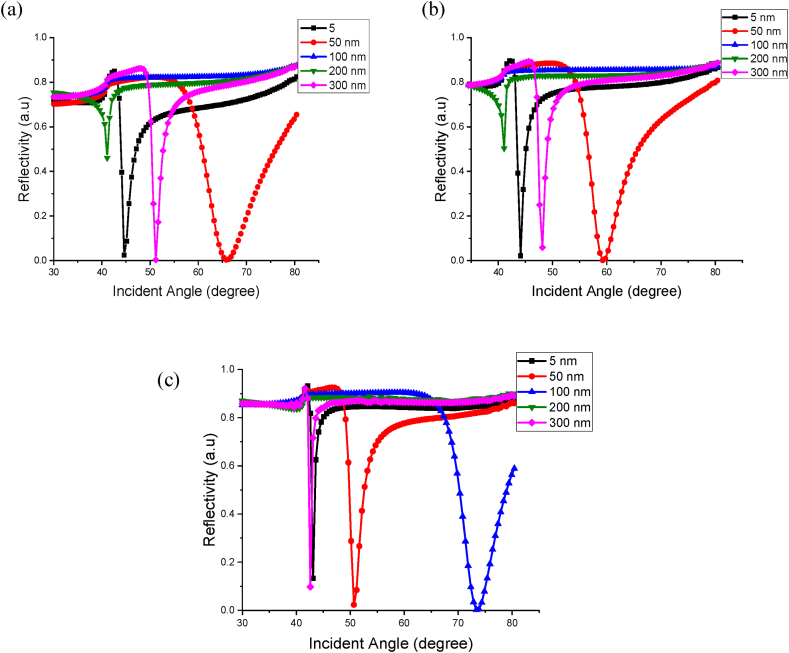
K-SPR reflectance curves obtained for the BK7/Cr-0.5 nm/Au-50 nm/ZnO/air system for ZnO thin film with 5 nm, 50 nm, 100 nm, 200 nm and 300 nm thickness at (a) 633 nm (b) 670 nm (c) 785 nm wavelength.

Numerous studies have been conducted to observe the optimum thickness of Au to be used in sensing applications in K-SPR, and it was found that 50 nm is the best thickness as it yields the smallest reflectivity compared to the other thicknesses [29,52]. Thus, for the first part of this paper, the thickness of Au used is 50 nm. To characterize the behaviour of nanohybrid Au (50 nm) - ZnO thin film at different thickness, the RI values are plotted versus incident angle using the results obtained from the FDTD simulation. The thickness of ZnO varied from 5, 50, 100, 200–300 nm at 633 nm, 670 nm and 785 nm wavelengths. The dielectric sensing medium is set to 1.0 for air. These simulations are conducted to observe the best thickness of ZnO for sensing purposes. Fig. 2 (a), (b) and (c) shows the K-SPR curve observed from the simulation at 633, 670 and 785 nm optical wavelength, respectively.

The results revealed that at 633 nm wavelength, the 300 nm thickness of ZnO yielded the best performance since it recorded the lowest $R_{\min}$ and FWHM value. As can be seen from the graph, the curve for 300 nm thickness has the lowest dip at $R_{\min}$ equal to 0.0031 a.u and the smallest FWHM value of 2.0224 °. It is important for $R_{\min}$ to have the lowest value since it shows a larger amount of energy transferred through the thin films to oscillate the surface plasmon, thus strengthening the energy of the surface plasmon wave [53]. For 100 nm thickness, the value of $R_{\min}$ and FWHM cannot be calculated since the K-SPR angle falls out of the scanning angular range. The same situation happened for ZnO-100 nm at 670 nm wavelength and ZnO-200 nm at 785 nm wavelength.

Next, the simulation is continued with a change of wavelength at 670 nm. The graph of reflectivity against incident angle is shown in Fig. 2 (b), and in this wavelength, the 5 nm ZnO thickness recorded the best K-SPR reflectance curve. At 5 nm, it recorded the smallest value of $R_{\min}$ at 0.0218 and FWHM = 1.4606 °. For the last part of varying ZnO thickness, the wavelength is changed to 785 nm, and the graph for this wavelength is illustrated in Fig. 2 (c). In this simulation, the 100 nm ZnO thickness recorded the smallest value of $R_{\min}$ = 0.0031, but the FWHM value recorded the highest value among the others. Thus, for this wavelength, 300 nm still recorded the best K-SPR reflectance curve for the sensing purpose. Hence, 5 nm and 300 nm are the potential thickness that can be used for the detection of uric acid. From these two thicknesses, 5 nm is chosen for the detection due to the availability of this sensor slide in our lab. Other than that, 5 nm is used due to the future experimental work that will be done based on this simulation results. The completed data for varying ZnO thickness of ZnO at 633 nm, 670 nm and 785 nm are shown in Table 2.

**Table 2:** Minimum reflectivity, K-SPR resonance angle and FWHM values for varying Zno thickness at 633 nm, 670 NM and 785 NM wavelengths.

Wavelength (nm)	Thickness of ZnO (nm)	Minimum Reflectivity (a.u)	K-SPR Resonance Angle (°)	FWHM (°)
633	5	0.0247	44.6218	2.4917
	50	0.0037	65.7983	12.6262
	100	–	–	–
	200	0.4603	41.0924	1.0231
	300	0.0031	51.1765	2.0224
670	5	0.0218	44.1176	1.4606
	50	0.8807	51.1765	7.7669
	100	–	–	–
	200	0.8300	51.1765	0.8911
	300	0.0586	48.1513	1.6332
785	5	0.1330	43.1092	0.7116
	50	0.0232	50.6723	2.5955
	100	0.0031	73.3613	7.8530
	200	–	–	–
	300	0.0974	42.6050	0.6401

**Fig. 3:**
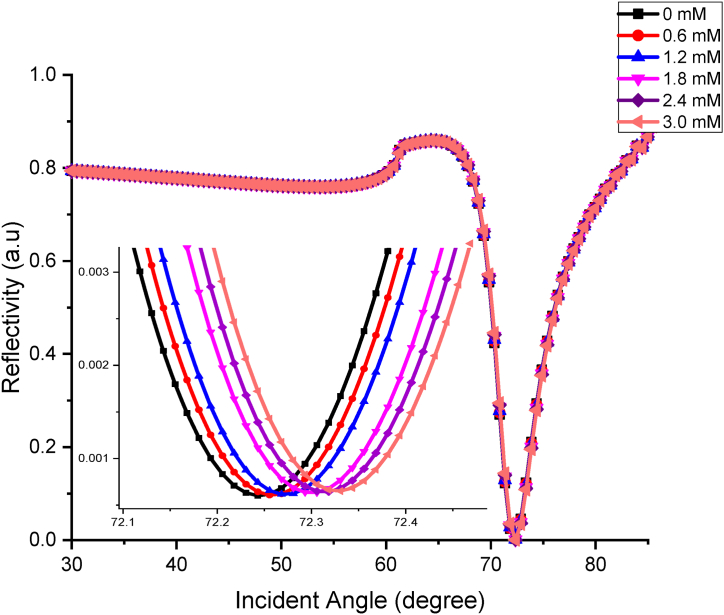
K-SPR reflectance curves obtained for the BK7/Cr-0.5 nm/Au-50 nm/ZnO-5 nm/uric acid at different concentrations at 670 nm wavelength.

The FDTD simulation is continued using the optimum thickness observed in the first part of the simulation with the change of optical parameter, background index and wavelength. The background index is changed corresponding to the different uric acid concentrations, while the wavelengths used are 670 nm and 785 nm. These wavelengths are used due to its availability in our laboratory equipment, Bionavis K-SPR Navi 200. The angle for the full sensing range used for this simulation is from 30 ° to 85 °. This simulation is done to study the ability of this sensor to detect the person with healthy and unhealthy concentrations of uric acid. The K-SPR reflectance curves observed for different concentrations of uric acid ranging from 0 mM to 3.0 mM at 670 nm is shown in Fig. 3. From the graph, it was found that the graph shifted to the right-hand side as the concentration of UA increased. This is because as the concentration of UA increases, the RI also increases, leading to a change in absorbance of the sensing layer. Thus, the reflectance value will also change [54]. The angle shifting of the K-SPR curve demonstrates that Au–ZnO layer at this wavelength has the potential to be used as uric acid sensor applications using the angular interrogation method.

**Fig. 4:**
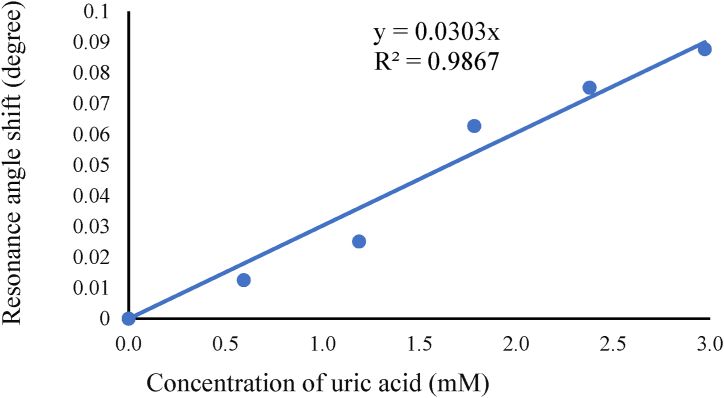
Resonance angle shift versus concentrations of uric acid at 670 nm wavelength.

Then, for the minimum reflectivity, $R_{\min}$ at which surface plasmon excitation occurs, the values range from 0.00060 to 0.00065, while the K-SPR angle falls in the range of 72.2431 ° to 72.3308 °. The average angle shift found in Fig. 3 is 0.05264 °. Small value of $R_{\min}$ obtained from this simulation indicates better performance of UA sensor and $R_{\min}$ of the reflected light happens as maximum light intensity is used to generate surface plasmon at resonance condition [55]. From the detection of UA at 670 nm wavelength graph, the FWHM values calculated are 5.1870 °, 5.2611 °, 5.1573 °, 5.2462 °, 5.1584 °, 5.3189 °, with respect to the increase in concentration from 0 mM to 3.0 mM. The 1.2 mM uric acid recorded the smallest FWHM value compared to the other concentrations observed. From the graph in Fig. 3, the angle shift versus concentration of UA is plotted and shown in Fig. 4. The angle shift, sensitivity and Q-factor data are tabulated in Table 3. The linear fitting line of the graph is shown in Eq. (4),

(4) $$\begin{array}{c} {\Delta \theta }_{SPR}=0.0303x\; \end{array}$$

where ${\Delta \theta }_{SPR}$ is the resonance angle shift and x is the concentration of uric acid. This equation indicates the sensitivity of the sensor is 0.0303° to any change in 1 mM concentration of uric acid. From the $R^{2}$ value, the performance of the sensor in terms of linearity is also high at 98.67 %.

**Table 3:** Change in resonance angle, sensitivity, Q-factor of Au-Zno based K-SPR uric acid sensor at 670 nm.

Concentration of uric acid (mM)	Resonance angle shift, ${\Delta \theta }_{SPR\;}$ (°)	Sensitivity (°/mM)	Q-factor (${\text{mM}}^{-1}$)
0.6	0.0125	0.0210	0.0040
1.2	0.0251	0.0211	0.0041
1.8	0.0627	0.0351	0.0067
2.4	0.0752	0.0316	0.0061
3.0	0.0877	0.0295	0.0055

**Fig. 5:**
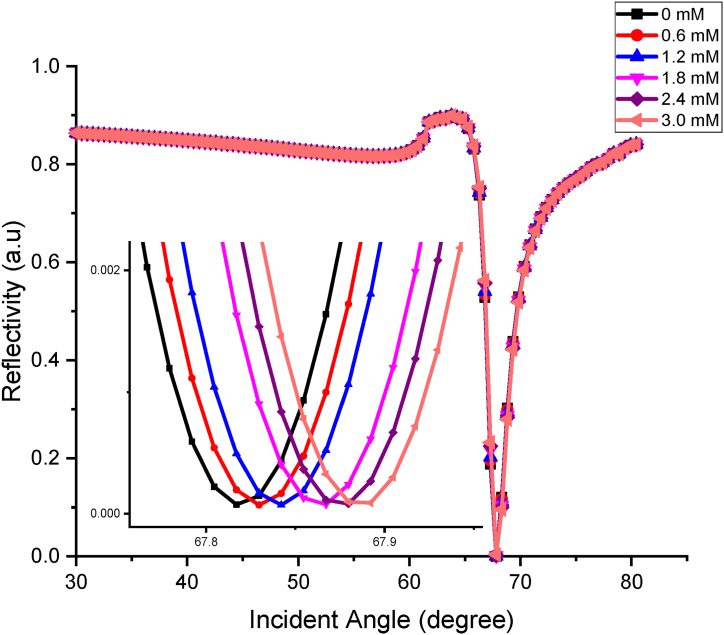
K-SPR reflectance curves for the BK7/Cr-0.5 nm/Au-50 nm/ZnO-5 nm/uric acid at different concentrations at 785 nm wavelength.

**Fig. 6:**
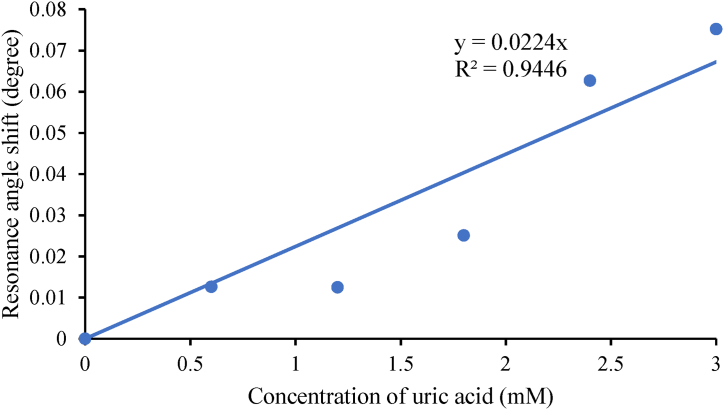
Resonance angle shift versus concentrations of uric acid at 785 nm wavelength.

The sensitivity of Au–ZnO at 670 nm is calculated using the formula in Eq. (2). This sensor's average sensitivity and Q-factor equal 0.028°/mM and 0.0053 ${\text{mM}}^{-1}$, respectively. Then, the simulation is continued by varying uric acid concentrations at 785 nm wavelengths. The K-SPR reflectance curve of this wavelength is shown in Fig. 5. As the concentration of uric acid increases, the graph is shifted to a higher angle, and the angle shift versus the concentration of UA is illustrated in Fig. 6. From the linear fitting graph, it is found that the linearity is equal to 94.46 % and compared to the 633 nm wavelength, the sensor has better linearity at 670 nm. Then, the $R_{\min}$ values range from 0.000075 a.u to 0.000090 a.u, and the K-SPR angles range from 67.8170 ° to 67.8922 ° with an average angle shift equal to 0.03762 °. The value of FWHM calculated from the 785 nm wavelength are 2.4876 °, 2.5077 °, 2.5091 °, 2.5268 °, 2.5434 °, 2.5331 ° corresponding to the concentration of UA of 0 mM–3.0 mM. The complete data for this wavelength is shown in Table 4. Compared with the 670 nm wavelength in terms of average sensitivity, the sensor has greater sensitivity at 670 nm than at 785 nm, 0.0193°/mM. This is due to a greater resonance angle shift that occurs when the concentration of uric acid increases at 670 nm wavelength compared to 785 nm. This accounts for the sensor's higher sensitivity at 670 nm. The practical implication of this heightened sensitivity is that the sensor at 670 nm can detect lower concentrations of uric acid with greater precision. While for the quality factor, the sensor has a better value of 0.0076 ${\text{mM}}^{-1}$ at 785 nm.

**Table 4:** Change in resonance angle, sensitivity, Q-factor of Au-Zno based K-SPR uric acid sensor at 785 nm.

Concentration of uric acid (mM)	Resonance angle shift, ${\Delta \theta }_{SPR\;}$ (degree)	Sensitivity (°/mM)	Q-factor (${\text{mM}}^{-1}$)
0.6	0.0126	0.0210	0.0084
1.2	0.0125	0.0104	0.0042
1.8	0.0251	0.0139	0.0055
2.4	0.0627	0.0261	0.0103
3.0	0.0752	0.0251	0.0099

**Fig. 7:**
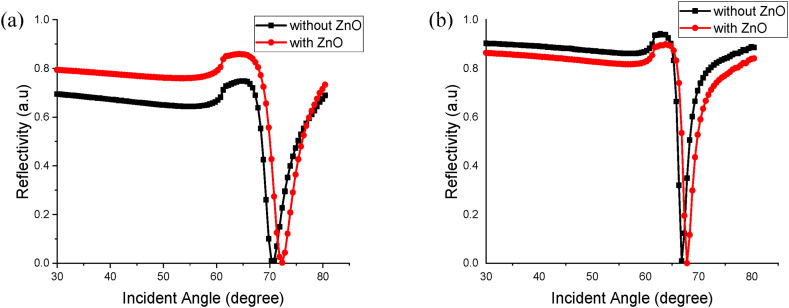
Comparison between the K-SPR Reflectance Curves of Au and hybrid Au/ZnO for Detection of Uric Acid at 0.6 mM at (a) 670 nm and (b) 785 nm.

For the last part of the simulation, the sensing performance of Au–ZnO is compared with the bare Au. Hence, the simulation is conducted using an optimum thickness of Au, 50 nm, to sense the lowest concentration of uric acid at 0.6 mM. This concentration is used due to the limit of detection in this paper which is 0.6 mM. The simulation is done by changing the background index corresponding to the concentration used and the ZnO layer is removed from the material data and script in the FDTD simulation. Both 670 nm and 785 nm wavelengths are used to observe bare Au's sensing performance. The simulation results are shown in Fig. 7 (a) and (b). As shown in the graph, both K-SPR curves for bare Au are shifted to the left hand-side due to a decrease in the material layers' RI. It is found that, the value of $R_{\min}$ at both wavelengths recorded a higher value than the ZnO-coated Au. At 670 nm, as shown in Fig. 7 (a), the $R_{\min}$ value for bare Au is 0.01004 a.u, while for ZnO-coated Au, the $R_{\min}$ is at 0.00061 a.u. Hence, it is proven that the ZnO layer has enhanced the performance of the sensor as it succeeded in decreasing the $R_{\min}$ by 0.00943 a.u and as mentioned before, lower $R_{\min}$ value is desired for good sensor performance.

Next, for 785 nm, shown in Fig. 7 (b), the $R_{\min}$ value recorded for bare Au is 0.00866 a.u, while from the previous simulation, the $R_{\min}$ value for ZnO-coated Au is 0.00007 a.u. By having ZnO on top of Au, the $R_{\min}$ value decreased by 0.00858 a.u. But, the FWHM values for bare Au at both wavelengths have a lower value than ZnO-coated Au. At 670 nm, the FWHM value recorded for bare Au is 4.7470 °, while the FWHM value for ZnO-coated Au is 5.2611 °. Then, at 785 nm, the FWHM value for bare Au in UA detection is 2.1860 ° and for ZnO-coated Au is 2.5077 °. The increment of FWHM when deposited ZnO on top of Au is due to the high RI value of ZnO widening the K-SPR resonance dip and causing the FWHM to increase [33]. The findings of this study are compared with those of a previous study on UA detection, which employed a different technique based on wavelength interrogation as shown in Table 5.

**Table 5:** Comparison of efficacy of SPR sensors with previous study for uric acid detection.

Material Layer	Mechanism Used	Sample	Concentration of Uric Acid	Sensitivity	Limit of Detection	Reference
AuNPs/Graphene Oxide	LSPR	Serum	0.01 mM–0.8 mM	0.0082 nm/μM (519 nm and 230 nm)	0.206 mM	[56]
AuNPs/Graphene Oxide	LSPR	Serum	0.01 mM–1.0 mM	2.1 %/mM (519 nm and 230 nm)	0.656 mM	[57]
AuNPs	LSPR	Serum	0.01 mM–0.8 mM	0.0131 nm/μM (520 nm and 527 nm)	0.280 mM	[58]
SMSMS/OFSs/AgNPs/CuO-NPs	LSPR	Urine	0.4 mM–10 mM	1.23 nm/mM (395 nm and 387 nm)	0.35 mM	[59]
Cr/Au/ ${\text{MoS}}_{2}$-Graphene	SPR	N/A	0 mM and 3.0 mM	85.25°/a.u (670 nm)	N/A	[12]
				74.61°/a.u (785 nm)		
Cr/Au/Zinc Oxide	SPR	Urine	0 mM–3.0 mM	0.028°/mM, 140.76°/a.u (670 nm)	0.6 mM	2023 (This work)
				0.0193°/mM, 100.40°/a.u (785 nm)		

From the comparison table, in terms of the detection range of uric acid (UA), our work has considered a wide range, making it suitable for future experimental applications. However, the difference in this sensing range is due to the lower concentration of UA found in serum samples, while a wider range of UA concentration is observed in urine. When compared with the work done by Agrawal et al. [59], detection range in our study is somewhat limited due to the inavailability of the refractive index data obtained from previous experimental work in that study. Regarding sensitivity, our study is unique as it is the only one based on angular interrogation at both 670 nm and 785 nm wavelengths, while other studies utilize wavelength interrogation. Therefore, comparing the sensor's performance in terms of sensitivity is challenging. The same scenario applies to the limit of detection. This paper aims to detect healthy UA concentrations as well as unhealthy concentrations at 3.0 mM. However, due to limitations in refractive index data, we can only test one concentration of unhealthy UA excretion from urine.

This study is also being compared with the previous study by N. A. Jamil et al. [12] that used the same sensing method, K-SPR, with a different material layer which is molybdenum disulfide (${\text{MoS}}_{2}$)-graphene. For comparison, the sensitivity of this work is recalculated using angle shift per change in RI. It was found that hybrid Au–ZnO sensor has better performance in terms of sensitivity at both wavelengths, 670 nm and 785 nm than ${\text{MoS}}_{2}$-graphene sensor for the detection of UA. Furthermore, the FWHM values observed for both wavelengths are lower than those reported in previous research. N. A. Jamil et al. measured FWHM values of 7.230° and 2.775° for 670 nm and 785 nm, respectively, while our study recorded FWHM values of 5.221° and 2.518° for 670 nm and 785 nm. This indicates that the hybrid Au–ZnO has better sensing performance than (${\text{MoS}}_{2}$)-graphene.

## 4 Conclusion

In conclusion, the Cr-0.5 nm/Au-50 nm/ZnO-5 nm layer is numerically studied for performance enhancement in the detection of uric acid. It was found that the sensor has the best performance at 670 nm, where the average sensitivity of the sensor is equal to 0.028°/mM, with Q-factor at 0.0053 ${\text{mM}}^{-1}$ and linearity at 98.67 %. By comparing with the bare Au for detection of uric acid, the ZnO layer has decreased the minimum reflectivity by 0.00943 a.u and 0.00858 a.u, at 670 nm and 785 nm, respectively. Therefore, ZnO has proven its ability as a sensing material for performance enhancements and is suitable to be used for future experimental work.

## Data availability

The data used in our study has not been deposited into a publicly available repository. The datasets used and/or analyzed during the current study will be made available from the corresponding author upon reasonable request.

## Impact and scope statement

In this work, we continue our research on the sensing of kidney health using angular interrogation of Kretschmann-based surface plasmon resonance operating at 670 and 785 nm optical wavelengths. Hybrid materials comprising of zinc oxide (ZnO) on top of gold (Au) sensor slides were used for the detection of uric acid. To our knowledge, this is the first time that hybrid Au–ZnO materials are being used for optical sensing of uric acid at these optical wavelengths. In this work, we used Finite Difference Time Doman (FDTD) simulation method to develop a plasmonic optical sensor to detect uric acid with molarity ranging from 0 to 3.0 mM. The main objective of this study is to observe the ability of the hybrid material as a sensing performance enhancer for differentiating between healthy and unhealthy uric acid levels based on the refractive index values from previous study. Upon exposure to 670 nm wavelength, the average sensitivity of this sensor was found to be 0.028°/mM with a linearity of 98.67 % and Q-factor value of 0.0053 mM^−1^. While at 785 nm, the average sensitivity is equal to 0.0193°/mM with slightly lower linearity at 94.46 % and Q-factor value of 0.0076 mM^−1^. The results have proven the ability of hybrid material Au–ZnO as a sensing performance enhancer for detecting uric acid when compared with bare Au and other hybrid materials.

## CRediT authorship contribution statement

**Siti Nasuha Mustaffa:** Writing - original draft, Visualization, Validation, Software, Methodology, Investigation, Formal analysis, Data curation. **Affa Rozana Abdul Rashid:** Supervision, Resources, Conceptualization. **Nadrah Md Yatim:** Supervision, Resources, Conceptualization. **Vatsala Pithaih:** Validation, Supervision, Software, Project administration, Methodology, Investigation, Formal analysis, Data curation, Conceptualization. **Nur Shahirah Sha'ari:** Writing - original draft, Validation, Methodology, Investigation, Formal analysis, Data curation. **Ahmad Razif Muhammad:** Writing - review & editing, Supervision, Conceptualization. **Azaham Abdul Rahman:** Resources. **Nur Akmar Jamil:** Writing - review & editing, Visualization, Supervision, Project administration, Investigation, Conceptualization. **P. Susthitha Menon:** Writing - review & editing, Writing - original draft, Visualization, Validation, Supervision, Software, Resources, Project administration, Methodology, Investigation, Funding acquisition, Formal analysis, Data curation, Conceptualization.

## Declaration of competing interest

The authors declare that they have no known competing financial interests or personal relationships that could have appeared to influence the work reported in this paper.
